# Report of a rare case and review of adult intestinal duplication at the opposite side of mesenteric margin

**DOI:** 10.1590/1516-3180.2017.0184030817

**Published:** 2017-12-07

**Authors:** Zhi-Hao Huang, Zi-Hao Wan, Vikash Vikash, Sindhu Vikash, Cong-Qing Jiang

**Affiliations:** I MD, MSc. Surgeon, Department of Colorectal and Anal Surgery, Wuhan University Zhongnan Hospital, Wuhan, Hubei Province, China.; II MD, MSc. Surgeon, Department of Orthopedic Surgery, Wuhan University Zhongnan Hospital, Wuhan, Hubei Province, China.; III MD, PhD. Physician, Department of Gastroenterology, Renmin Hospital of Wuhan University, Wuhan, Hubei Province, China.; IV MD, PhD. Physician, Department of Medicine, Shaheed Mohtarma Benazir Bhutto Medical University, Larkana, Sindh, Pakistan.; V MD, PhD. Surgeon, Department of Colorectal and Anal Surgery, Wuhan University Zhongnan Hospital, Wuhan, Hubei Province, China.

**Keywords:** Adult, Intestines Congenital abnormalities

## Abstract

**CONTEXT:**

To study the previously discovered clinical entity of adult intestinal duplication and its treatment, and propose an extension to its existing classification.

**CASE REPORT:**

We report the case of an adult male with abdominal pain, constipation and vomiting. This patient underwent surgical separation of adhesions, reduction of torsion and intestinal decompression. Postoperative pathological findings confirmed the rare diagnosis of intestinal duplication.

**CONCLUSION:**

Adult intestinal duplication is quite rare. Its clinical manifestations are nonspecific. From this finding of intestinal duplication originating at the opposite side of the mesenteric margin, a further extension of the existing anatomical classification is proposed.

## INTRODUCTION

Digestive tract duplication is a rare congenital malformation that can involve any segment of the gastrointestinal tract.^1^ It is common during the fetal, neonatal and pediatric period. Clinicians are prone to misdiagnose it because of the nonspecific clinical signs. Although the diagnostic significance of enteroscopic and radionuclide imaging has been reported, there are limitations to these imaging techniques because of the diverse symptoms and complexity of anatomical sites of this disease. At present, surgical resection is still the main treatment. The results from a systematic search of the literature in the main database is shown in Table 1. We aimed to compare and contrast our patient’s clinical data and associated previous studies, in order to further discuss the diagnosis and treatment options.

**Table 1:** Summary of adult patients with small intestinal duplications as found in the United States National Institutes of Health’s National Library of Medicine (PubMed) database as of July 20, 2017

Database	Search strategies				Papers found	Related papers
MEDLINE (via PubMed)	(((“Adult”[Mesh]) AND “Intestine, Small”[Mesh]) AND “Congenital Abnormalities”[Mesh]) AND duplication[Title/Abstract]				40	20
**Study (year)**	**Age (years)/gender**	**Presenting features**	**Surgical treatment**	**Form**	**Size (cm)**	**Malignancy**
Ho (2012)^3^	25/male	Abdominal pain	Yes	Tubular	NR	No
Vivier et al. (2013)^4^	32/female	Routine control examination	Yes	Cystic	2	No
Handra-Luca et al. (2013)^5^	65/male	Routine control examination	Yes	Tubular	8	Yes
Barbosa et al. (2015)^6^	36/male	Abdominal pain, vomiting	Yes	Cystic	NR	No
Yu et al. (2014)^7^	24/female	Abdominal pain	Yes	Cystic	28 × 20	No
Li et al. (2013)^8^	25/male	Abdominal pain, weight loss	Yes	Tubular	15.5 × 4	No
Nussbaum et al. (2014)^9^	51/male	Abdominal pain, bloody stools	Yes	Tubular	9.5 × 2	Yes
Kim et al. (2014)^10^	19/female	Abdominal pain	Yes	Cystic	2,5	No
Park et al. (2014)^11^	36/female	Abdominal pain	Yes	Cystic	12	No
Martínez-Alcala et al. (2014)^12^	37/female	Abdominal pain, vomiting	Yes	Cystic	5	No
Furuya et al. (2012)^13^	70/female	Abdominal pain	Yes	Cystic	3	Yes
Miloudi et al. (2012)^14^	26/male	Abdominal pain, vomiting	Yes	Cystic	4	No
Palacios et al. (2013)^15^	35/female	Routine control examination	Yes	Cystic	1.3	No
Antaki et al. (2013)^16^	21/male	Abdominal pain	Yes	Cystic	NR	No
Kusnierz et al. (2014)^17^	31/female	Abdominal pain, nausea, lack of appetite	Yes	Cystic	2.9 × 2.6	No
Cheng et al. (2014)^18^	49/male	Gastrointestinal bleeding, melena passage	Yes	Cystic	NR	No
Kurien et al. (2014)^19^	NR/female	Abdominal pain	Yes	Cystic	NR	No
Kachi et al. (2016)^20^	64/female	Abdominal pain	Yes	Cystic	4	No
Gupta et al. (2016)^21^	20/male	Constipation, vomiting	Yes	Cystic	NR	No
Inoue et al. (2016)^22^	56/male	Appetite loss, palpitations, orthostatic syncope, hematochezia	Yes	Cystic	NR	No

NR = not reported.

## CASE REPORT

A 53-year-old male patient presented to our emergency department with the main complaint of abdominal pain and constipation for two days. His past medical and family histories were unremarkable. He had been smoking for 20 years (10-15 cigarettes a day). He denied having any allergies or previous illicit drug or alcohol use.

**Figure 1:**
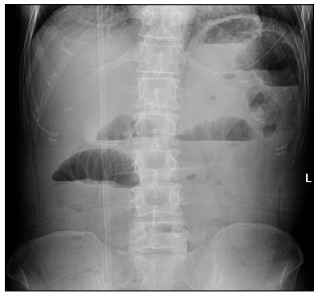
Abdominal radiograph: multiple gas-liquid planes in the intestinal gut.

**Figure 2:**
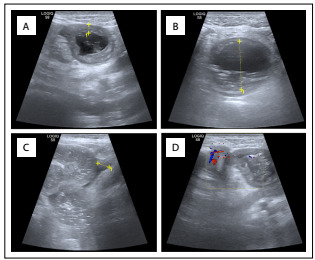
Abdominal ultrasonography: (A) A distended bowel can be seen below the umbilicus, and peristalsis is not apparent. The wall of the tube is thickened, and the lumen at the beginning of its expansion is compressed. (B) A dark area is seen between the intestines. (C) The intestinal wall is raised into the lumen, and the two intestinal tubes share the same wall of the bowel canal. (D) A small amount of colored blood flow signal can be seen on the wall of the dilated intestinal tube.

**Figure 3:**
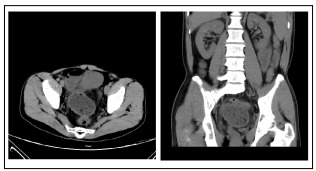
Abdominal and pelvic computed tomography examination: A large cystic mass with a clear boundary can be seen.

On physical examination, the patient’s body temperature was 36.8 °C, the abdomen was soft and slightly bulged, there were no apparent bowel movements, no apparent mass on palpation, negative shifting dullness, no auscultated sounds of an overactive bowel and a digital rectal examination was negative for any mass or blood. Laboratory tests suggested slight elevation in white blood cell count. An abdominal radiograph revealed multiple air-fluid levels in the intestinal gut (Figure 1). Abdominal ultrasonography showed a dilated bowel sharing a common continuous wall with the distal segment (Figure 2). Computed tomography (CT) revealed thickening of the small intestinal wall and cystic intestinal dilation proximal to stenosis with clear margins (Figure 3).

The patient was given symptomatic treatment consisting of antibiotics, antacids, antispasmodics and enema. After one day, he presented with noticeable abdominal swelling with unbearable abdominal pain, total abdominal tenderness, rebound tenderness and bowel sounds that had decreased in intensity.

**Figure 4:**
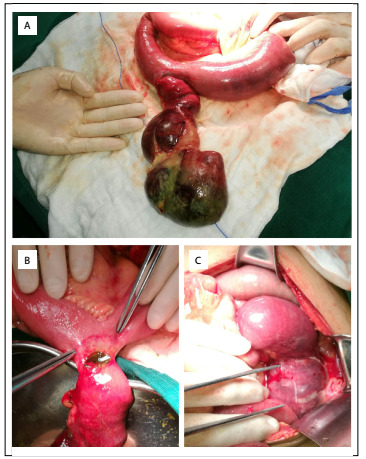
Intraoperative findings (gross specimens): (A) The mass was generally ischemic, with darker color; the surface was covered with pus and no perforation was found; the proximal segment of the small intestine was dilated and hypertrophic, with inflammatory edema and punctate ischemia. (B) The basement wall was relatively weak, and the border of the normal bowel was clear. (C) Twisted sticky joints at the base of the mass compressed the adjacent normal intestinal canal.

Subsequently, exploratory laparotomy was performed. On surgical exploration, around 100 ml of transparent ascites fluid and torsion of a dilated small bowel loop with surrounding edema were noticed. Moreover, a large cystic mass was detected hanging from the wall of the small intestine into the pelvic cavity, which had led to formation of inflammatory adhesions with adnexa. The adhesions were then bluntly separated, and the mass was found to be located at around 150 cm distally to the Treitz ligament. The mass was ischemic, dark in color, and covered with pus. The basal wall of the mass was weaker than the healthy loops around it, and the basal diameter was around 4.5 cm. After relieving the twisted mass and the surrounding small intestine, we noticed inflammatory edema, and dilation of the proximal intestinal wall. Several adhesive masses with punctate ischemia and inflammation, 20 cm proximally to the lesion, was also noted (Figure 4).

**Figure 5:**
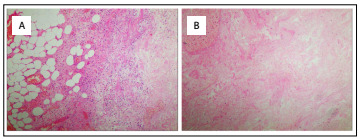
Pathological findings: (A) Small bowel cystic lesions: hemorrhage and necrosis were observed in the intestinal wall, and mesenteric vascular dilatation and congestion were observed, which was consistent with bleeding and necrotic alterations in the volvulus (hematoxylin and eosin staining, magnification x 100). (B) Intestinal ischemia and inflammatory tissue: examination of intestinal wall tissue showed presence of a mucosal layer of chronic inflammatory cell infiltration, with submucosal edema and thickening, while the smooth muscle layer was not abnormal (hematoxylin and eosin staining, magnification x 100).

We decided to loop-out the jejunum approximately 35 cm proximally and 5 cm distally to the base of the mass. After resection of about 40 cm of bowel, which included the mass and the ischemic small intestine, the remaining bowel segments were anastomosed using a stapler. An intraoperative diagnosis of intestinal duplication, obstruction and torsion of the small intestine was made. The pathological findings are shown in Figure 5.

Postoperatively, the patient was kept under parenteral nutrition. He recovered well and began eating nine days after the surgery. He was discharged fifteen days after the surgery, and no sign of recurrence was observed after three months of follow-up.

## DISCUSSION

Digestive tract duplication is more common in men and tends to occur most frequently in the small intestine. It characteristically arises from the mesenteric border of the bowel.^2^ Although several cases (Table 1)^3^^,^^4^^,^^5^^,^^6^^,^^7^^,^^8^^,^^9^^,^^10^^,^^11^^,^^12^^,^^13^^,^^14^^,^^15^^,^^16^^,^^17^^,^^18^^,^^19^^,^^20^^,^^21^^,^^22^ of adult intestinal duplication have been reported, the present case was unique, in that the mass emerged from the opposite side of the mesenteric margin, which made intestinal obstruction and torsion more likely to occur.

The broadly accepted classification of intestinal duplication consists of five types: intestinal membrane type, intestinal wall cyst type, extra-intestinal cyst type, extra-intestinal tubular type and solitary type.^23^ The prevalence of gastrointestinal duplication varies, but it is more common in the ileum and ileocolic segment. Intestinal duplication is usually cystic or tubular in nature and often continuous with the regular bowel wall, and it shares the muscle and mucosal layer.^24^

We hypothesized that the course of this case would have begun with repeated duplication of the jejunum, thus resulting in repeated intestinal edema, enlargement and inflammatory changes. Subsequently, under gravitational traction, the inflamed bowel would have been able to twist easily. Abnormal duplication would then have occurred and the adjacent bowel walls would have aligned and compressed each other.

The clinical presentation of intestinal duplication depends on the structure, size and site of the lesion in relation to the surrounding structures. Because of nonspecific signs and symptoms, it is often misdiagnosed as cases of other causes of an acute abdomen at the acute stage, or is labeled as Meckel’s diverticulum at the chronic or asymptomatic stages. Meckel’s diverticulum has a separate blood supply, while intestinal duplication shares a blood supply with the surrounding intestinal tract. Moreover, intestinal duplication is characterized by well-developed smooth muscle, which is absent from intestinal diverticula.

At present, the types of imaging that are suggested are ultrasonography, gastrointestinal radiography, computed tomography, radionuclide imaging and magnetic resonance imaging.^25^^,^^26^ Over recent years, comparatively more cases have been diagnosed during the fetal period, and this has been attributed to widespread use of obstetric ultrasound scanning. Ultrasonography reveals a separate echo structure, double wall sign and a shared bowel wall, which points towards the potential abnormality. Therefore, ultrasonography is preferred over other types of imaging. It has also been reported that ^99m^Tc radionuclide imaging has diagnostic value for cases of suspected gastric mucosal ectopy.^27^ However, in our case, no gastric mucosal ectopy was seen in the sectioned specimen.

In this disease, surgical treatment is the preferred option. The choice of surgical procedures depends on multiple factors, such as the location, type and relationship with the surrounding tissue.

## CONCLUSION

The classification of digestive tract duplication is diverse. This is the first case report on small intestinal duplication on the opposite side of the mesenteric margin. It potentially extends the anatomical classification of intestinal duplication.

## References

[1] Blank G, Konigsrainer A, Sipos B, Ladurner R (2012). Adenocarcinoma arising in a cystic duplication of the small bowel: case report and review of literature. World J Surg Oncol.

[2] Keramidas DC, Demetriades DM (1996). Total tubular duplication of the colon and distal ileum combined with transmesenteric hernia: surgical management and long-term-results. Eur J Pediatr Surg.

[3] Ho YC (2012). Total colorectal and terminal ileal duplication presenting as intussusception and intestinal obstruction. World J Gastroenterol.

[4] Vivier PH, Beurdeley M, Bachy B, Aguilella C, Ickowicz V, Lemoine F (2013). Ileal duplication. Diagn Interv Imaging.

[5] Handra-Luca A (2013). Adult ileal duplication revealed by a colon adenoma. Surg Today.

[6] Barbosa L, Soares C, Povoa AA, Maciel JP (2015). Ileal duplication: an unusual cause of intestinal obstruction in adult life. BMJ Case Rep.

[7] Yu Y, Wu JS, Ke ZW (2014). Giant chylous cyst and ileal duplication in a young adult. Singapore Med J.

[8] Li BL, Huang X, Zheng CJ, Zhou JL, Zhao YP (2013). Ileal duplication mimicking intestinal intussusception: a congenital condition rarely reported in adult. World J Gastroenterol.

[9] Nussbaum DP, Bhattacharya SD, Jiang X, Cardona DM, Strickler JH, Blazer DG (2014). Gastroesophageal heterotopia and HER2/neu overexpression in an adenocarcinoma arising from a small bowel duplication. Arch Pathol Lab Med.

[10] Kim HS, Sung JY, Park WS, Kim YW (2014). An ileal duplication cyst manifested as an ileocolic intussusception in an adult. Turk J Gastroenterol.

[11] Park JY, Her KH, Kim BS, Maeng YH (2014). A completely isolated intestinal duplication cyst mimicking ovarian cyst torsion in an adult. World J Gastroenterol.

[12] Martinez-Alcala Garcia F, Perez Pozo JM, Martinez-Alcala Garcia A, Ciria Avila JA, Martinez Alcala F (2014). [Duodenal duplication cyst and its endoscopic resolution]. Gastroenterol Hepatol.

[13] Furuya K, Hada M, Sugai H, Miyasaka Y, Nakagomi H, Oyama T (2012). Gastrointestinal stromal tumor arising in an ileal duplication: report of a case. Surg Today.

[14] Miloudi N, Mzoughi Z, Ben Abid S, Sabbagh S, Arfa N, Khalfallah MT (2012). [Duodenal duplication treated surgically]. La Tunisie medicale.

[15] Palacios A, De Vera M, Martinez-Escoriza JC (2013). Prenatal sonographic findings of duodenal duplication: case report. J Clin Ultrasound.

[16] Antaki N, Abboud D, Lemmers A, Antaki F, Deviere J (2013). Acute recurrent pancreatitis secondary to the rare association of a duodenal duplication cyst and a pancreas divisum. Clin Res Hepatol Gastroenterol.

[17] Kusnierz K, Pilch-Kowalczyk J, Gruszczynska K, Baron J, Lucyga M, Lampe P (2014). A duodenal duplication cyst manifested by duodenojejunal intussusception and chronic pancreatitis. Surgery.

[18] Cheng CL, Liu NJ, Yu MC (2014). Intraluminal tubular duodenal duplication with bleeding. Clin Gastroenterol Hepatol.

[19] Kurien RT, Chowdhury SD, Unnikrishnan LS, Simon EG, Dutta AK, Mahanta K (2014). Endoscopic treatment of a duodenal duplication cyst. Endoscopy.

[20] Kachi A, Haddad F, Geagea A, Tohmeh MJ (2016). Unusual case of jejunal duplication in a 64-year-old female. J Med Liban.

[21] Gupta A, Chakaravarthi K, Pattnaik B, Kaman L (2016). Duplication cyst of ileum presenting as acute intestinal obstruction in an adult. BMJ Case Rep.

[22] Inoue K, Sakiyama T, Setoyama K, Iwashita Y, Saito S, Hanada N (2016). [Jejunal duplication in an adult presenting with hematochezia]. Nihon Shokakibyo Gakkai Zasshi.

[23] Fuyou H, Hong Y, Yan S (1997). The diagnosis and classification of intestinal duplication. Chinese Journal of Pediatric Surgery.

[24] Sinha A, Ojha S, Sarin YK (2006). Completely isolated, noncontiguous duplication cyst. Eur J Pediatr Surg.

[25] Nadatani Y, Watanabe T, Sugawa T, Eguchi S, Shimada S, Otani K (2016). Double-balloon endoscopy WAS effective in diagnosing small intestinal duplication: a case report. SpringerPlus.

[26] Ogino H, Ochiai T, Nakamura N, Yoshimura D, Kabemura T, Kusumoto T (2008). Duplication cyst of the small intestine found by double-balloon endoscopy: a case report. World J Gastroenterol.

[27] Schwesinger WH, Croom RD, Habibian MR (1975). Diagnosis of an enteric duplication with pertechnetate 99mTc scanning. Ann Surg.

